# Normalized Ploidy Following 20 Consecutive Blastocysts with Chromosomal Error: Healthy 46, XY Pregnancy with IVF after Intraovarian Injection of Autologous Enriched Platelet-derived Growth Factors

**DOI:** 10.22088/IJMCM.BUMS.8.1.84

**Published:** 2019-05-15

**Authors:** E. Scott Sills, Natalie S. Rickers, Channel S. Svid, J. M. Rickers, Samuel H. Wood

**Affiliations:** 1 *Gen 5 Fertility; La Jolla, California, USA.*; 2 *Office for Reproductive Research, Center for Advanced Genetics; La Jolla, California, USA.*; 3 *Department of Obstetrics and Gynecology, Palomar Medical Center; Escondido, California, USA.*

**Keywords:** Reproductive outcome, cytokines, ploidy, blastocyst

## Abstract

One explanation for why downstream gonadotropin protocol changes during IVF commonly arrive too late to have significant effects is that embryo development actually begins during oogenesis. Thus, efforts to modify the chromosomal status of blastocysts must address the ovarian milieu well in advance of follicular recruitment. A 42 year old woman with primary infertility of 3 year duration attended with her partner. Five previous IVF cycles had produced 20 embryos, but all had genetic abnormalities and no embryo transfer was performed. Karyotypes and all lab tests were normal for both partners. 3 months before her IVF here, she received isolated platelet-derived growth factors injected into both ovaries as a cell-free, enriched substrate. Genetic assessments were via whole genome amplification and DNA tagmentation and PCR adapter sequences. Comprehensive chromosomal screening was carried out by dual-indexed sequencing of pooled libraries on the MiSeq™ platform. From this IVF cycle one euploid 46, XY blastocyst was produced and vitrified on the day of trophectoderm biopsy. 9 days after frozen embryo transfer, serum human chorionic gonadotropin was 250 mIU/ml and a transvaginal ultrasound at 6 week gestation confirmed a single intrauterine pregnancy with fetal heart at 153/min. A healthy male infant was delivered by c-section at 39 weeks' gestation. While cellular and molecular events directing the oocyte-to-embryo transition are incompletely characterized, processes related to ovarian stem cell differentiation, mitochondrial dynamics, and mRNA storage, translation, and degradation likely are relevant. It appears that intraovarian application of autologous platelet-derived growth factors, when used before IVF, can impact oocyte integrity and facilitate euploid blastocyst development. Although research on intraovarian injection of autologous activated platelet rich plasma has already shown improved quantitative IVF responses, this is the first description of qualitative improvements in embryo genetics after intraovarian injection of autologous platelet-derived growth factors.

Ovarian aging leads to decreases in both quantity and quality of eggs, negatively impacting implantation rate and increasing frequency of miscarriage. Any identified genetic imbalance generally carries a negative prognosis, and is increasingly used as criteria to de-select blastocysts for transfer or cryopreservation. The clinical value of preimplantation embryo data has caused disagreement among experts (1). However, it has even been suggested that mosaicism or ploidy error detected during extended *in vitro* culture may not always be an absolute barrier to a subsequent healthy pregnancy (2, 3). Yet female age and embryo genetics represent the main drivers of clinical treatment decisions before and during IVF. A better understanding of ovarian senescence, gamete maturation, and production of chromosomally competent blastocysts remain key areas for progress in the field.

While cellular and molecular events directing the oocyte-to-embryo transition are incompletely characterized at present, processes related to ovarian stem cell differentiation, mitochondrial dynamics, and mRNA storage, translation, and degradation are likely relevant (4). This view would support the hypothesis that embryo development actually begins during oogenesis, involving complex early signaling events leading to a healthy, competent zygote (5). This model may help explain why downstream adjustments in IVF gonadotropin protocols simply arrive too late to accomplish anything meaningful, especially for older infertility patients with the poorest prognosis.

Here we describe pre-IVF injection of platelet-derived growth factors into ovaries which had previously failed to produce any eggs capable of generating a euploid blastocyst. While limited research already has linked ovarian platelet rich plasma (PRP) treatment with quantitative improvement in serum AMH and increased oocyte yield with IVF (6-8), this is the first report of embryo euploidy being achieved after ovarian injection of platelet-derived growth factors.

## Case presentation

This 42- year old Caucasian nulligravida (BMI =20 m/kg^2^) with primary infertility of 3 years duration attended with her partner. Although the couple had undergone five IVF cycles at two different facilities, none of the 20 embryos generated during treatment were transferred due to genetic abnormalities identified during pre-implantation genetic screening (Table 1). Review of previous clinical records showed consistent, adequate responses to numerous gonadotropin stimulation regimes; no cycles were cancelled (or converted to intrauterine insemination (IUI)) because of poor follicular recruitment. Serum anti-Müllerian hormone (AMH) here was 0.97 ng/ml, consistent with ovarian reserve appropriate for chronological age. Both partners were nonsmokers in excellent health. Standard G-band karyotypes and Recombine™ carrier screening panel (Reprogenetics; Livingston NJ USA) identified no mutations or deletions for either partner. Semen analysis was unremarkable and sperm chromatin analysis returned a DNA fragmentation index of 9%.

Prior to embarking on her next (sixth) IVF cycle, at this center the patient received autologous PRP injection to both ovaries under ultrasound guidance at this center (8). Three months later, controlled ovarian hyperstimulation with 150 IU/d recombinant follicle-stimulating hormone (rec-FSH) + 150 IU/d human menopausal gonadotropin (hMG) commenced incorporating a gonadotropin-releasing hormone (GnRH) -antagonist (9), selected after review of prior cycle data. While six eggs were retrieved and five advanced to the blastocyst stage, unfortunately all were aneuploid, and therefore embryo transfer was not performed. The following month, after transvaginal ultrasound exam confirmed normal appearing ovaries andreturn of menses post-IVF, a second bilateral intraovarian injection was performed—this time with isolated platelet-derived growth factors prepared as an enriched, cell-free substrate (Neokine™; Chicago USA) using a technique similar to previously reported methods (10, 11).

A three, month interval elapsed before commencing her final IVF; for this cycle a GnRH-antagonist was again utilized but gonadotropin doses were adjusted (Table 1). Uniquely, this treatment action resulted in one euploid blastocyst which was cryopreserved on day of trophectoderm (TE) biopsy. Genetic assessments at our clinic and at the outside facilities were uniformly from blastocyst TE biopsy, which provided material for whole genome amplification and DNA tagmentation and PCR of adapter sequences. Next, comprehensive chromosomal screening was carried out by dual-indexed sequencing of pooled libraries on the MiSeq™ platform (Illumina; San Diego USA).

Nine days after blastocyst thaw and transfer, the patient’s serum human chorionic gonadotropin (hCG) was 250 mIU/ml and transvaginal ultrasound at six weeks’ gestation confirmed a single intrauterine pregnancy with fetal heart at 153/min. The patient underwent uncomplicated c-section delivery at 39 weeks' gestation had a healthy male infant (3459 g). Mother and child continue to do well at home. Written informed consent for treatment and unrestricted permission to publish/present these data in a non-identifiable manner were obtained by the patient.

**Table 1:** Summary of selected IVF treatment features and blastocyst ploidy status before and after bilateral intraovarian injection of autologous platelet rich plasma and platelet-derived growth factors

Date		Protocol	Oocytes (*n*)	PGS data
2016	JUN	225FSH+75hMG, GnRH- ant; 10K IU hCG	12	47,XY+22; 44,XX-11-21; 44,XY-4-17
	JUL	leuprolide downreg> 225FSH+150hMG, 25u hGH; 10K IU hCG	7	47,XX+19; 46,XY-1+5 (mos 6,7,8,9,10,11,14,17,21,22)
	AUG	leuprolide downreg> 225FSH+150hMG, 25u hGH; 10K IU hCG	13	46,X+19; 47,XY+2; 45,XY-22; 45,XY-19; 46,XX+9-18; 47,XX+1
	DEC	450FSH+150hMG, GnRH-ant; 10K IU hCG+80u leuprolide	14	44,XX-4-6; 47,XY+21; 44,XY-13-19; 47,XX+7; 48,XY+1+18; 47,XY+13; 44,X-17
2017	MAY	225FSH+150hMG, 25u hGH, GnRH-ant; 10K IU hCG	10	45,XX-21; 44,XY-14-17
2018	JAN	❶PRP		
	APR	150FSH+150hMG, GnRH- ant; 2500IU hCG+80u leuprolide	5	47,XX+19; 45,XY-22; 46,XX+19-22; 46,XX-2+20
	MAY	❷EnPLAF		
	AUG	75FSH+300hMG, GnRH-ant; 2500IU hCG+80u leuprolide	6	47,XY+19; 43,XX-13-15-22; 45,XX-8; 40,XX-4-13-14-16-18-19; 46,XY

## Discussion

For many infertility patients, the intractable challenge of embryo aneuploidy is an unwelcome and serious detour on the journey to pregnancy. Developing a meaningful way to enhance the genetic status of embryos is a long-pursued aim of reproductive biology research, representing a therapeutic discovery having an impact difficulty to overstate. Here, we present the first description of pre-IVF use of autologous platelet-derived growth factors for an advanced age fertility patient, where ploidy rescue and a healthy singleton pregnancy resulted. Particularly notable is the correction of a three-year pattern of genetic error identified in consecutive blastocysts. Aspects of this case may begin to offer an answer to the question “What, if anything, can be done to modify the chromosomal constituency of embryos from IVF?”

How cytokines produced and isolated from activated platelets might impact chromosomal competency during oogenesis remains speculative. The autologous cell-free substrate used here is known to be comprised of cytokines, chemokines, and growth factors including platelet-derived growth factor (PDGF), stromal cell derived factor 1 (SDF-1), and hepatocyte growth factor (HGF). These molecular signals initiate recruitment, proliferation, and activation of fibroblasts, neutrophils, monocytes, and other cells central to wound healing. Within the adult human ovary, these mediators likewise would be expected to regulate angiogenesis and tissue perfusion which might be an independent way towards ooplasm improvement (12).

Placing autologous cytokines within ovarian tissue could have facilitated the higher AMH, and improved blastocyst ploidy through at least two mechanisms. One possibility is that any follicles recruited and oocytes obtained after intraovarian injection of these growth factors had always been potentially present—latent, but then stimulated. Alternatively, upon contacting ovarian tissue, these platelet growth factors engaged with uncommitted ovarian stem cells, supplying molecular signals needed to promote differentiation to develop *de novo* eggs. *In vitro* observations of platelet-rich plasma effects on growth and survival of isolated early human follicles tend to support such theories, as development and survival rates of preantral follicle in PRP-supplemented culture media have been found to be significantly higher than where this supplementation is lacking (13).

It is also known that aged eggs contain fewer mitochondria, have impaired fertilization, and poor embryonic development—perhaps in part due to altered mitochondrial dynamics (14). A strong connection here seems plausible, as a relatively high mtDNA copy number has been reported among aneuploid embryos (15). If one result of ovarian treatment with platelet-derived growth factors has simply improved ooplasm quality, then a subsequent mitochondrial reset could be another process by which ploidy rescue of blastocysts works.

**Fig. 1:**
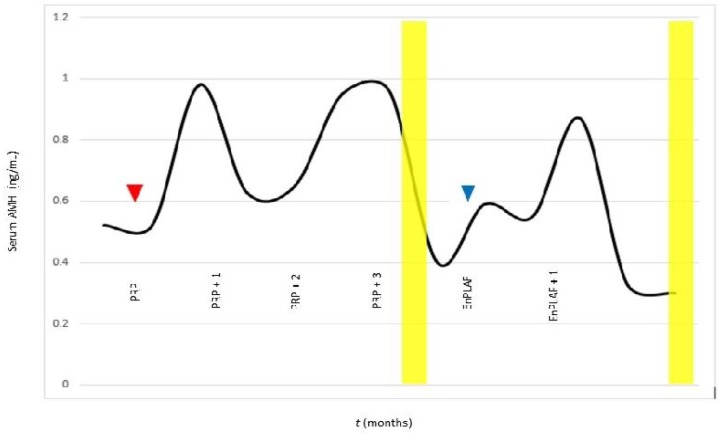
Measured fluctuations in ovarian reserve. Serum AMH was measured as a function of bilateral intraovarian injection of autologous platelet rich plasma (red arrow); cell-free platelet derived growth factors assessmrny is represented by blue arrow. Stimulated IVF cycles and oocyte retrievals associated with each intervention are also depicted (yellow bands). From the second cycle, one 46, XY blastocyst was produced which was subsequently transferred and resulted in a single (ongoing) intrauterine pregnancy

Several cautions are warranted regarding interpreting this research. Numerous interventions occurred when various independent clinics ran multiple IVF cycles, even though the laboratory technique used to screen embryo genetics was uniform throughout. The choice of stimulation protocols is not trivial as gonadotropin type, dose, and duration (with or without hGH) is considered influential for final oocyte development and IVF outcome (15). Thus, it must be admitted that the observed “correction” of embryo ploidy error cannot be exclusively ascribed to intraovarian injection of platelet-derived growth factors. But if administration of platelet derived growth factors contributed only to measured changes in serum AMH, this itself would be a welcome response because low AMH levels are regarded as an independent risk factor for embryo aneuploidy among fertility patients at age ≥35 years (17). Also, it was impossible to know exactly when IVF should occur, to take best advantage of any such beneficial AMH “lift”. This means the timing of our IVF may not have been optimal, and additional euploid blastocysts might have been generated if a different sequence was followed. And finally, this IVF patient underwent two experimental interventions here—conventional PRP, and then isolated (cell free) platelet derived growth factors. In our experience, some IVF patients who are refractory to intraovarian PRP injection respond better to isolated platelet-derived growth factors prepared as a cell-free, enriched substrate. But because this patient received both treatments in succession (Figure 1), the possibility that one intraovarian injection “prepared” for the next cannot be excluded.

Additional investigation with ovarian elements (including ovarian stem cells) is likely to clarify signaling pathways involved in oocyte replenishment and follicular development, thereby providing potential techniques to make IVF successful even for older patients with very low ovarian reserve (18). The ovarian germline stem cell niche and its probable regulatory mechanisms are currently being explored, yielding valuable insights and scope for the prevention and treatment of ovarian senescence (19). Because the patient described here had demonstrated sufficient reserve to complete multiple oocyte collections and create blastocysts *in vitro*, her interest in this investigational treatment was somewhat atypical. Previously, those receiving ovarian injection of autologous platelet-derived growth factors had been dismal prognosis IVF patients with numerous cycle cancellations, consistently showed undetectable serum AMH, or were “ovarian failures” of unknown etiology. Early research on improved quantitative IVF responses following intraovarian PRP based on small numbers of poor-prognosis patients appear encouraging (7,8), but qualitative improvement in embryo genetics after intraovarian injection of autologous platelet-derived growth factors has not been reported until now.

## Conflict of interest

ESS holds a provisional U.S. patent for process & treatment using ovarian platelet rich plasma.
